# Flame-Retardant Battery Pack Case Design for Delaying Thermal Runaway: A CFD and Experimental Study

**DOI:** 10.3390/ma18245605

**Published:** 2025-12-13

**Authors:** Hyun Soo Kim, Mingoo Cho, Dongwook Lee, Changyeon Lee, Jaewoong Kim, Sungwook Kang

**Affiliations:** 1 Jinju Center, Dongnam Technology Application Division, Korea Institute of Industrial Technology, Jinju-si 52845, Republic of Korea; hyun0702@pusan.ac.kr (H.S.K.); cmg0142@pusan.ac.kr (M.C.); 2 School of Mechanical Engineering, Pusan National University, Busan 46241, Republic of Korea; 3 F&S Company, Hwaseong-si 18576, Republic of Korea; dongwook@fnsolution.kr; 4 R&D Center, Daejoo Kores Company, Wanju-gun 55316, Republic of Korea; cyeonlee@daejookc.com; 5 Purpose Built Mobility Group, Korea Institute of Industrial Technology, Gwangju 61012, Republic of Korea; 6 Department of Smart Ocean Mobility Engineering, Changwon National University, Changwon-si 51140, Republic of Korea

**Keywords:** thermal runaway, battery pack case (BPC), flame-retardant design, CFD and experiments, electric vehicle safety

## Abstract

Thermal runaway (TR) in lithium-ion batteries presents a significant safety hazard for electric vehicles (EVs), often resulting in fire or explosion. Mitigating TR requires thermal-protection strategies capable of delaying or suppressing heat propagation within battery pack cases (BPCs). This study proposes a flame-retardant BPC design and evaluates its effectiveness through a combined approach using CFD-based thermal analysis and multiscale experimental validation. In the CFD model, a heat-source temperature of 1107 °C was applied to simulate the thermal load during TR, together with a coolant flow rate of 17 L/min. Material-level verification was conducted through high temperature specimen tests, in which flame-retardant pads were heated to a target of 1100 °C with an allowable tolerance of ±10% for 5 min; the unheated (backside) temperature remained below 160 °C. Full-scale assessment involved heating the BPC upper case at temperatures exceeding 500 °C for 10 min, where the backside temperature remained below 150 °C. Module-level TR experiments further confirmed that the flame-retardant layer reduced the external temperature from 240–260 °C to below 150 °C. The results demonstrate that the proposed design effectively delays thermal penetration and maintains external safety thresholds, offering practical guidelines for developing safer EV battery systems.

## 1. Introduction

The global demand for electric vehicles (EVs) has increased rapidly over the past decade, driven by advances in power electronics, cooling technologies, and battery systems [1]. At the core of this transition are lithium-ion batteries (LIBs), which offer high energy density, long cycle life, and excellent power performance compared with conventional energy-storage technologies [2,3]. However, LIBs also pose significant safety challenges, among which thermal runaway (TR) is recognized as one of the most critical hazards because it can lead to fires or explosions [4,5,6]. Once initiated in a single cell, TR may propagate to neighboring cells and escalate to the module or pack level, as evidenced by well-documented EV fire incidents involving manufacturers such as Tesla, BMW, and Hyundai [7].

Extensive research has been devoted to understanding TR mechanisms, particularly at the cell level. Early modeling and experimental studies established multidimensional thermal-abuse frameworks and highlighted the role of internal structure and jelly-roll configuration in governing TR evolution [8]. Subsequent investigations have examined the formation of internal short circuits and the spatio-temporal development of TR using three-dimensional electro-thermal models [9,10]. Multiscale calorimetry and X-ray imaging studies have further clarified TR onset and progression, demonstrating dependencies on SOC, chemistry, and abuse conditions [11,12].

However, most TR studies have remained limited to the cell scale because cell-level tests are easier to control experimentally, require lower thermal/structural loads, and avoid the substantial safety risks, cost, and infrastructure demands associated with pack-level TR testing. In addition, computational resources required for pack-level CFD or coupled multiphysics simulations are significantly higher, which have historically constrained large-scale investigations. As a result, although cell-scale studies provide essential mechanistic insight, they are insufficient for evaluating thermal-barrier performance and flame-retardant behavior within full pack enclosures.

From a system perspective, the nature of combustion products and toxic gases released during LIB fires has emerged as a critical concern. Experimental analyses have shown that TR events generate hazardous species such as hydrogen fluoride and other toxic fluorinated gases, underscoring the need for effective containment and ventilation strategies [9,13]. These findings emphasize that safety solutions must not only control temperature and mechanical integrity but also address the chemical toxicity associated with battery failures.

To mitigate TR and maintain safe operating temperatures, a variety of battery thermal-management systems (BTMSs) have been proposed. Numerical and experimental studies have explored open-cell aluminum-foam heat sinks [14], enhanced forced-air systems with optimized foam geometries [15], and cross-linked cold-plate designs tailored to high-power packs [16]. Hybrid PCM–liquid-cooling systems have also been examined to improve transient and steady-state performance [17]. While these strategies provide improved temperature regulation, they often require increased design complexity, packaging volume, or manufacturing cost, which poses challenges for mass-produced EV platforms [18,19]. In parallel, accurate thermophysical property data have been reported to improve CFD-based simulations of cell and module behavior [12,20].

Material-based TR mitigation methods have gained attention as passive alternatives to active cooling. Prior studies have demonstrated that enclosure design and inter-cell spacing influence TR propagation [21], and thermochemical heat-storage materials have been proposed to absorb exothermic energy [22]. Mechanical–compressible foams have been evaluated for their dual function as structural buffers and thermal barriers [23]. Interstitial thermal-barrier materials have also been tested for containment of side-wall rupture at the module scale [24]. These studies highlight the promise of passive flame-retardant approaches.

However, not all flame-retardant materials are suitable for EV applications. Polyurethane (PU) foams suppress TR effectively in pouch-cell modules [21], but fire-science studies report that PU combustion releases highly toxic gases such as hydrogen cyanide and carbon monoxide [25], limiting its automotive applicability. Recent pack-level TR studies have therefore shifted toward safer materials and pack-architecture-coupled mitigation strategies, including the role of cathode chemistry [5] and integration of experimental and high-fidelity modeling approaches [3,4,26].

Pack-scale TR behavior has further been explored using combined experimental and numerical frameworks. Investigations of flame ejection and particle behavior in module-scale TR tests highlight the importance of venting strategies and enclosure stiffness [2,24]. Large-format pack studies integrating CFD with experimental characterization have assessed TR propagation scenarios and enclosure modifications [3,4,21,26]. Deep learning-assisted CFD models have also been proposed to improve predictive accuracy for flame spread and gas dispersion in complex pack geometries [27].

Beyond thermal considerations, the structural safety of battery systems plays a critical role. Reviews of current-collector materials have linked mechanical reliability to electro-thermal performance [28]. Impact studies have characterized deformation modes at the cell level [29], while pack-level PSD-based vibration durability analyses have benchmarked fatigue behavior under service conditions [10,30]. Lightweight structural designs and topology-optimized enclosures have also been proposed [31,32,33], and cooling-channel layouts have been incorporated into structural simulations for coupled analysis [34]. Standardization efforts have reviewed test procedures and post-crash fire safety requirements [13,35], while application-specific works have evaluated sealing performance [36] and crashworthiness of carbon-fiber enclosures [37].

Despite these advances, critical research gaps remain. Most TR mitigation studies rely on cell- or module-level evaluation, and pack-enclosure-scale validation of flame-retardant concepts is still limited. Few studies directly examine how flame-retardant materials behave within a realistic pack enclosure during TR, and even fewer connect material-level data to system-level thermal modeling within a unified methodology.

Therefore, this study proposes a flame-retardant enclosure concept for EV battery pack cases using silicone-based pads as a passive thermal-protection layer. Silicone provides high thermal stability and reduced toxic-gas generation compared with PU, making it a strong candidate for EV BPC integration. To bridge the existing research gap between cell-scale understanding and pack-enclosure-scale validation, this work adopts a multi-layered evaluation strategy—linking specimen-level characterization of silicone pads, component-level furnace testing of a BPC upper case, and pack-level CFD analysis under TR conditions. This material–component–system approach provides an integrated assessment of silicone-based flame-retardant performance and offers practical guidance for future EV battery-safety standards and industrial pack designs.

## 2. Materials and Methods

In this study, both computational fluid dynamics (CFD) analysis and experimental validation were conducted to assess the TR delay performance of an EV BPC. The CFD simulations focused on the heat transfer and cooling characteristics of the upper case to investigate the heat diffusion delay mechanism. Subsequently, specimen-level heat resistance tests and upper-case fire resistance tests were performed to validate the numerical results.

### 2.1. Model Description

The CFD analysis was performed using ANSYS Fluent 2024 R2 (ANSYS Inc., Canonsburg, PA, USA). The simulation model was constructed based on the full geometry of the EV BPC, configured to replicate the internal flow characteristics and thermal diffusion behavior. Battery packs contain complex internal structures—including cooling channels, module support frames, ribs, and fastening components—that significantly increase mesh count when fully modeled.

To maintain computational efficiency, geometrical features with minimal influence on thermal behavior were removed. Fine details such as bolt holes, small filets, micro-ribs inside cooling channels, embossing features, and minor surface irregularities were simplified, whereas major structural components affecting flow resistance and heat transfer were retained. Small elements such as bolt-head geometries were also omitted because they do not meaningfully alter the global temperature distribution or structural boundary conditions relevant to flame-retardant evaluation.

**Figure 1:**
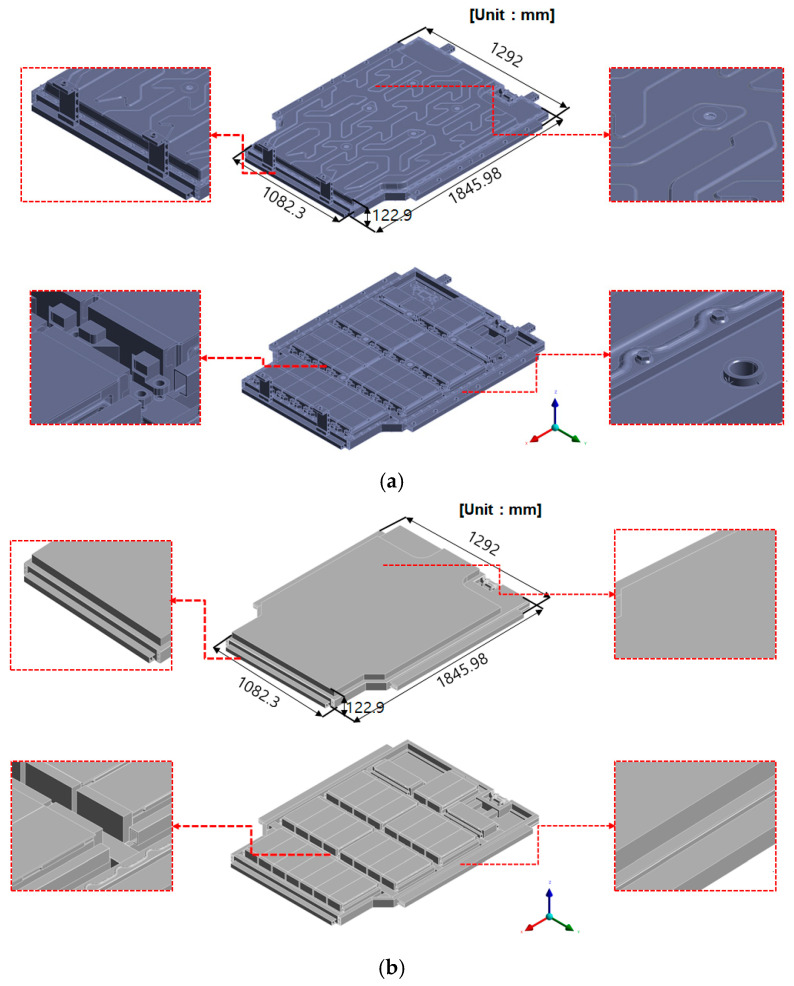
Geometry of the battery pack case used for CFD modeling: (**a**) Original model with detailed internal structures. (**b**) Simplified full-pack model after geometric reduction for CFD analysis.

This simplification strategy improved meshing quality and reduced computational cost by eliminating unnecessary high-aspect-ratio cells, while preserving the dominant thermal–mechanical behavior of the BPC. Figure 1 compares the original and simplified models.

**Figure 2:**
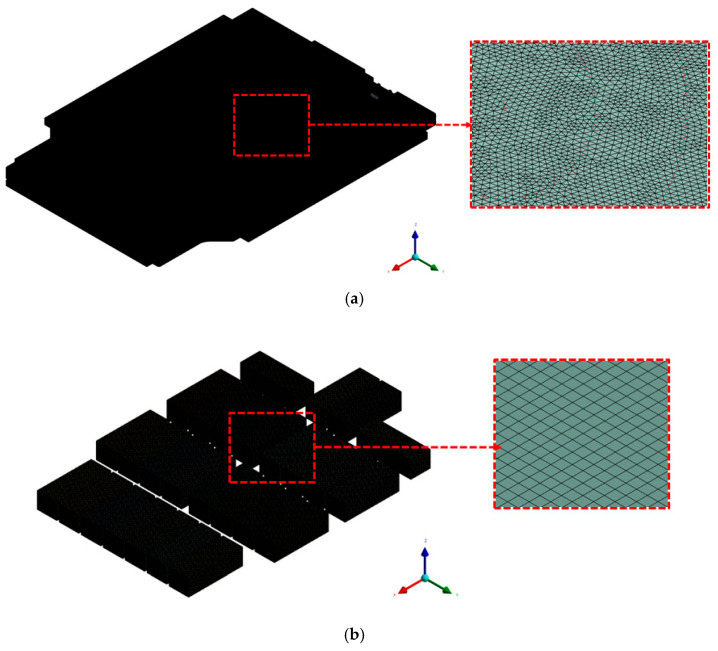
Computational mesh of the full battery pack case model: (**a**) Tetrahedral mesh applied to complex regions such as cooling channels with locally refined cells. (**b**) Hexahedral mesh applied to simple geometries, illustrating the hybrid meshing strategy.

Mesh generation was performed using ANSYS Fluent Mesher, and the overall mesh configuration, including local refinement around the cooling channels and structural boundaries, is shown in Figure 2. The final mesh consisted of approximately 16,561,219 nodes and 46,263,204 elements, and representative mesh specifications for the cooling-channel, module, and wall regions are summarized in Table 1. A hybrid meshing strategy was applied, combining tetrahedral elements in geometrically complex regions (e.g., cooling channels and curved surfaces) with hexahedral elements in simpler and more uniform regions. This mixed-element configuration allowed accurate resolution of critical flow zones while maintaining overall computational efficiency. Mesh quality satisfied the recommended skewness and orthogonal-quality criteria for transient CFD simulations. The maximum cell skewness was 0.89, which is below the generally accepted upper limit of 0.95 in ANSYS Fluent. No elements violated orthogonal-quality requirements. Additionally, grid independence was confirmed using the grid convergence index (GCI) method [38].

### 2.2. Boundary Conditions

**Figure 3:**
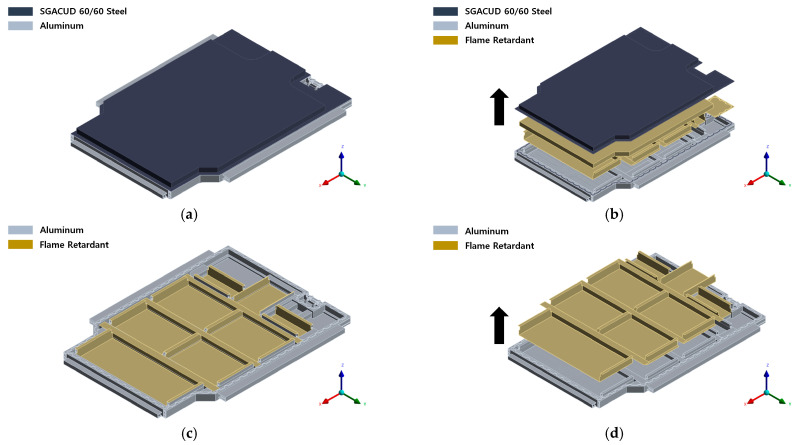
Geometry and exploded views of the battery pack case: (**a**) Full model with SGZCUD 60/60 steel, aluminum, and flame-retardant layers. (**b**) Exploded view of the full model. (**c**) Partial model highlighting flame-retardant placement. (**d**) Exploded view of the partial model.

The simulations were conducted using a simplified model based on the EV BPC’s full geometry. To construct the CFD model, several geometrical simplifications were applied to improve computational efficiency. Small features such as bolt holes, embossing patterns, ribs, fastening grooves, filets, and minor surface irregularities were removed because these elements have minimal influence on large-scale heat transfer and temperature distribution during thermal runaway. In addition, the detailed embossing patterns of the cooling-channel surface were replaced with an equivalent uniform wall surface to avoid excessive mesh refinement. These simplifications preserved the global thermal behavior of the pack while reducing the computational cell count by more than an order of magnitude. This approach ensured computational efficiency while accurately capturing the heat-transfer and flow characteristics. Figure 3 shows the geometry and exploded views of both the full and partial models. The primary materials used in the analysis included SGZCUD 60 steel (Hyundai Steel, Dangjin-si, Chungcheongnam-do, Republic of Korea), aluminum alloys such as AA6061P, AA6082S-T6, AA3003, and A6N01S-T6 (all supplied by Daejoo Kores Co., Ltd., Wanju-gun, Jeollabuk-do, Republic of Korea), PA6-GF30 polymer (Lotte Chemical, Seoul, Republic of Korea), and a silicone-based flame-retardant layer and EPDM pad (both supplied by F&S Company, Hwaseong-si, Gyeonggi-do, Republic of Korea). The silicone-based flame retardant was strategically positioned within the module and along the case walls to suppress heat transfer and delay TR propagation [39].

**Figure 4:**
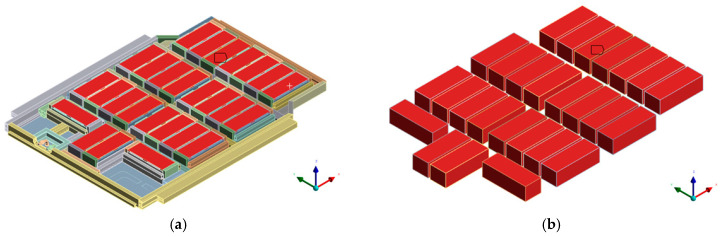
Thermal boundary conditions applied to the battery pack case: (**a**) Full pack model showing the cells highlighted in red, which were defined as heat-source regions. (**b**) Isolated view of the selected cell, where a 1107 °C heat source was imposed to represent thermal-runaway ignition.

As shown in Figure 4, the heating conditions for the battery cells were established by applying localized heat sources to each module unit. Under TR conditions, a localized heat source of 1107 °C was applied to the entire target module to represent the thermal environment after TR initiation. Although the typical temperature range for TR onset in LIBs is 120–200 °C, this value serves only as a reference. In this analysis, the initiation process itself was not simulated; instead, the extreme post-ignition conditions were simplified and imposed directly. This approach is consistent with the TR peak temperature range of 800–1100 °C reported in previous studies [40,41,42].

**Figure 5:**
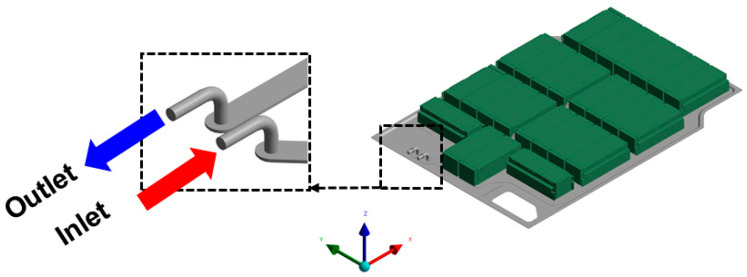
Coolant-side boundary condition with LCC-10: inlet flow rate of 17 L/min and inlet temperature of 26.9 °C (300 K); schematic of inlet/outlet ports of the cooling channel.

The cooling conditions were based on the LCC-10 coolant used in actual EV systems, as shown in Figure 5 [43]. The inlet was set to a flow rate of 17 L/min and a temperature of 26.9 °C (300 K). According to Tesla’s Model 3 Owner’s Manual, the BMS monitors cell temperatures during both driving and stationary conditions and activates the cooling pump as needed [44]. Therefore, this study assumes that active coolant circulation occurs during TR events.

The key material properties used in this study are summarized in Table 2 and Table 3, which include the density, specific heat, viscosity, and thermal conductivity of the coolant and BPC components, respectively [19,45,46,47,48,49,50]. These values were sourced from the literature to ensure the accuracy of the simulations. The numerical analysis focused on precisely evaluating the heat transfer and flow characteristics within the BPC, modeling the baseline design condition with the silicone-based flame retardant applied. The governing equations used during the simulation include the continuity, momentum, and energy equations, which incorporate conduction and convection as the heat transfer mechanisms. Radiative heat transfer was excluded from the scope of this study.

### 2.3. Numerical Method

The numerical analysis concentrated on investigating the heat transfer and flow characteristics within the EV BPC. The baseline model was developed under design conditions that incorporated a silicone-based flame retardant. The governing equations used in the analysis included continuity, momentum, and energy equations while focusing on conduction and convection as the main heat transfer mechanisms. Radiative heat transfer was excluded from the scope of this study.

The realizable k–ε model was employed for turbulence analysis. This model provides greater accuracy than the standard k–ε model and can more realistically represent the effects of rotation and curvature, making it widely used in thermal-fluid analyses related to battery cooling and thermal runaway [50].

Pressure–velocity coupling was achieved using the SIMPLE algorithm [51]. For discretization, a second-order scheme was applied to the pressure equation, and a second-order upwind scheme was applied to the momentum and energy equations, respectively [52,53]. These numerical schemes ensure convergence stability and computational accuracy in complex thermal-fluid problems.

Computations were conducted in steady-state mode. Although TR is inherently time-dependent, this study involved a large-scale model containing tens of millions of grid cells, for which computational efficiency and convergence stability were prioritized. Consequently, the analysis focused on the average heat transfer behavior rather than local transient variations. This approach enabled effective assessment of the thermal diffusion delay characteristics at the BPC level.

### 2.4. Experimental Setup (Specimen-Level Test)

**Figure 6:**
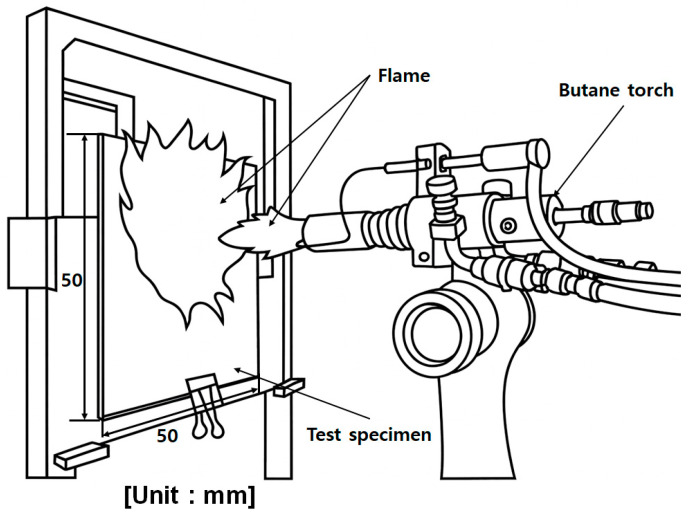
Schematic of the specimen-level flame test setup: 50 mm× 50 mm specimen exposed to a direct flame in accordance with UL 94 V-0 flammability evaluation standard.

In this study, specimen-level flame tests were performed to quantitatively evaluate the thermal insulation and flame-retardant performance of a silicone-based flame-retardant material applied to EV battery pack cases (BPCs). Square specimens (50 mm × 50 mm, 1.1 mm thickness) were fabricated to reproduce real-world application conditions. As shown in Figure 6, each specimen was mounted on a jig inside a standardized methane furnace, and the testing procedure followed the UL 94 V-0 vertical flame method, as summarized in Table 4.

The furnace temperature was maintained at 1100 °C (±10%), and the total flame exposure duration was 5 min, which is significantly more severe than the <50 s exposure required for UL 94 V-0. The distance between the burner nozzle and the front surface of the specimen was fixed at 95 mm, while the rear-surface temperature was measured at a distance of 150 mm using a T-type thermocouple (±0.5 °C accuracy).

Repeated tests under identical conditions were conducted to ensure reproducibility. During the experiment, the following flame-response characteristics were monitored:

(i) Ignition behavior;
(ii) Combustion duration after flame removal;
(iii) Charring depth and combustion-induced surface damage;
(iv) Structural deformation.

To quantitatively evaluate thermal insulation performance, additional specimens were instrumented with rear-surface thermocouples to record temperature histories during flame exposure. These specimen-level results provided fundamental validation data for assessing the thermal durability of the silicone-based flame-retardant material. The results were also used as reference input for subsequent BPC-level testing (Section 2.5) and CFD analyses (Section 3.1).

**Figure 7:**
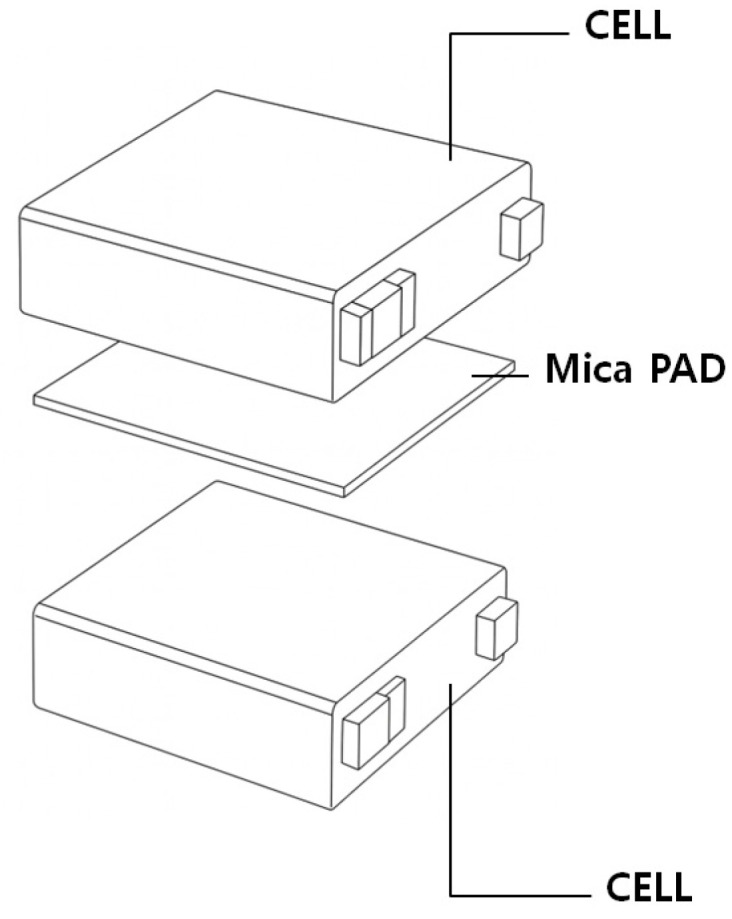
Example configuration of a mica-based thermal barrier pad placed between battery cells, as commonly used in prior studies to locally delay thermal runaway propagation.

Furthermore, Figure 7 presents an example from prior studies in which mica-based thermal barrier pads were placed between battery cells to locally delay thermal runaway propagation. Such prior approaches motivated the selection of a silicone-based flame-retardant material in this study, supported by commercial product data confirming UL 94 V-0 certification and high-temperature stability up to approximately 350 °C [55]. In addition, recent studies have reported that localized cell temperatures exceeding ≈160 °C can trigger thermal runaway onset [56], reinforcing the need to maintain rear-surface temperatures below this threshold. The specimen-level test results were therefore essential in demonstrating whether the proposed material can effectively suppress heat transfer beneath this critical limit.

### 2.5. Experimental Setup (Upper-Case-Level Test)

This test was conducted on an actual EV battery pack upper case to evaluate its fire-resistance performance under external fire conditions. The experiment followed the GB/T 31467.3-2015 [57] standard, which, similar to ISO 12405-3:2014 [58] and UN ECE Regulation No. 100 [54], specifies procedures for direct flame exposure using a fuel burner.

**Figure 8:**
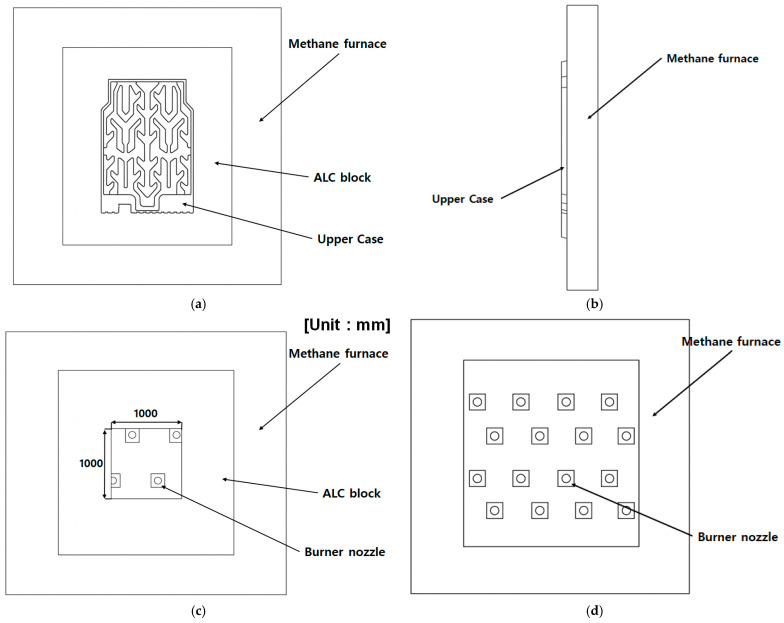
Schematic of the upper-case-level flame test setup in a vertical methane furnace: (**a**) Front view of the upper case positioned with ALC block inside the furnace. (**b**) Side view of the installed upper case. (**c**) Detailed view of the ALC block opening with a size of 1000 mm × 1000 mm. (**d**) Front view of the burner nozzle arrangement in the vertical furnace.

The experimental setup, depicted in Figure 8, included a vertical methane furnace. Inside the furnace, multiple burner nozzles were arranged vertically to directly heat both the upper and side surfaces of the battery pack upper case. The specimen was positioned in the center of an opening surrounded by autoclaved lightweight concrete (ALC) blocks. This opening was machined to a size of 1000 mm × 1000 mm to allow full insertion of the upper case, ensuring that the specimen was subjected to flame loads similar to actual operating conditions.

Six thermocouples (TC1–TC6) were attached to the unexposed surface to measure the temperature distribution during the test. The furnace temperature was monitored and controlled using multiple thermocouples and a control system. The objective was to maintain identical heating conditions for at least 10 min after the furnace temperature exceeded 500 °C and to evaluate whether the maximum temperature on the unexposed surface remained below 150 °C. During the test, the furnace temperature exceeded 500 °C approximately five minutes after ignition and stabilized around 530 °C for 10 min.

This procedure enabled a quantitative validation of the upper case’s fire-resistance performance with the silicone-based flame retardant, confirming that effective thermal insulation and flame-blocking performance were achieved through the combination of structural design and material properties. Along with the specimen-level experiment in Section 2.4, this test empirically demonstrated that the performance validated at the material level was consistently maintained in the full-scale system.

## 3. Results

This section presents the numerical and experimental findings obtained to evaluate the thermal-runaway (TR) delay performance of the proposed flame-retardant EV battery pack case (BPC). A three-stage validation framework was adopted to systematically verify the effectiveness of the silicone-based flame-retardant design across multiple scales.

First, CFD simulations were conducted to compare the heat-transfer characteristics of the BPC with and without the silicone-based flame-retardant pad, thereby identifying the mechanisms responsible for heat-flux suppression and delayed thermal diffusion (Section 3.1).

Next, specimen-level flame tests were performed to quantitatively characterize the thermal insulation behavior of the silicone-based material under direct, high-temperature exposure (Section 3.2).

Finally, a full-scale upper-case furnace test was carried out to experimentally confirm whether the flame-retardant design is capable of limiting structural temperature rise and delaying TR-like heat propagation under pack-level boundary conditions (Section 3.3).

Through this multi-stage validation approach, consistent trends were observed at the material, component, and system levels. When the CFD predictions were compared with experimental measurements, the results collectively demonstrated that incorporating the silicone-based flame-retardant pad significantly reduces heat transfer into the case structure, thereby enhancing TR delay performance at the pack-enclosure scale.

### 3.1. CFD Simulation Results

**Figure 9:**
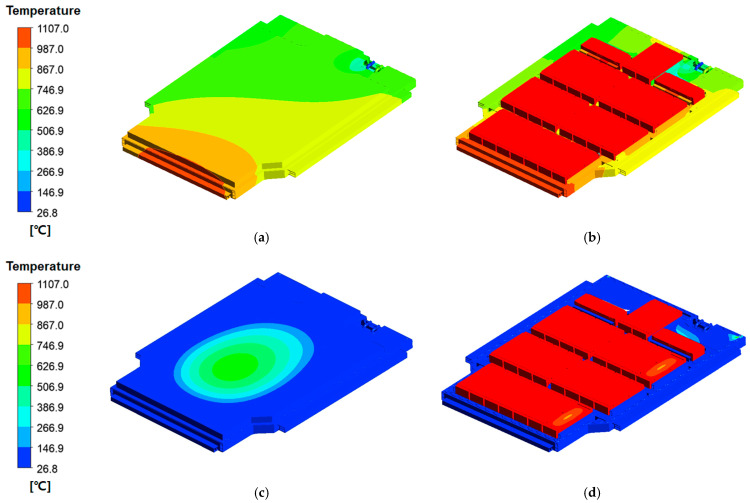
Temperature distribution of the battery pack case obtained from CFD analysis under identical thermal-runaway heat-source conditions (1107 °C): (**a**) outer surface without the flame-retardant pad, showing localized heating near the flame-exposed region due to the absence of thermal insulation; (**b**) internal structure without the flame-retardant pad, exhibiting unobstructed heat transfer around the module wall and rib regions; (**c**) outer surface with the silicone-based flame-retardant pad applied, where heat dispersion is significantly attenuated and peripheral temperatures remain low; (**d**) internal structure with the flame-retardant pad applied, indicating effectively suppressed heat transfer around the module wall and rib regions, demonstrating the enhanced thermal-protective performance of the flame-retardant design.

Figure 9 compares the temperature distribution of the battery pack case under identical thermal-runaway heat-source conditions (1107 °C), with and without the silicone-based flame-retardant pad applied.

Figure 9a presents the outer-surface temperature distribution without the flame-retardant pad. In this configuration, intense localized heating occurs near the flame-exposed region, and heat is rapidly conducted across the upper wall due to the absence of thermal insulation. As a result, the peripheral regions also experience elevated temperatures, indicating that heat dispersion is insufficient to limit the spread of thermal energy across the case surface.

Figure 9b shows the corresponding internal temperature field. Without the flame-retardant pad, heat transfer around the module walls and rib structures is largely unmitigated, producing extensive high-temperature zones within the supporting frame. This behavior reflects direct thermal conduction from the heat source to the structural components, increasing the likelihood of thermally induced deformation or material degradation.

In contrast, Figure 9c illustrates the outer-surface temperature distribution with the silicone-based flame-retardant pad. The results demonstrate a markedly different behavior: heat dispersion is significantly attenuated, and temperature decay toward the periphery is rapid. The outer wall remains at substantially lower temperatures because the flame-retardant pad introduces an additional thermal-resistance layer, reducing both the magnitude and the spatial extent of heat propagation. This behavior confirms that the pad acts as an effective thermal barrier, limiting external case heating even under TR-equivalent conditions.

Figure 9d shows the internal structure with the flame-retardant pad applied. Compared with Figure 9b, the high-temperature zones around the module walls and rib regions are substantially reduced. Heat transfer into the structural components is suppressed, and the thermal gradients are significantly smoother. This indicates that the silicone-based material not only delays heat diffusion but also protects structural members from direct thermal loading. Such suppression of internal heat transfer is essential for preventing structural softening and maintaining enclosure integrity during TR-induced thermal events.

Overall, the comparative results in Figure 9 clearly show that the silicone-based flame-retardant pad reduces heat conduction paths, weakens thermal coupling between cells and structural components, and significantly delays thermal diffusion into the case. This behavior supports the conclusion that the proposed flame-retardant design enhances the thermal-protective performance of the EV BPC under severe TR-like conditions. Although a fixed heat-source temperature of 1107 °C was applied to maintain consistent CFD boundary conditions, additional sensitivity checks confirmed that ±50 °C variations in the heat-source temperature changed the peak upper-case temperature by only 4–7%. This indicates that the TR-delay performance of the flame-retardant layer is governed primarily by its material properties rather than small fluctuations in heat-source severity.

**Figure 10:**
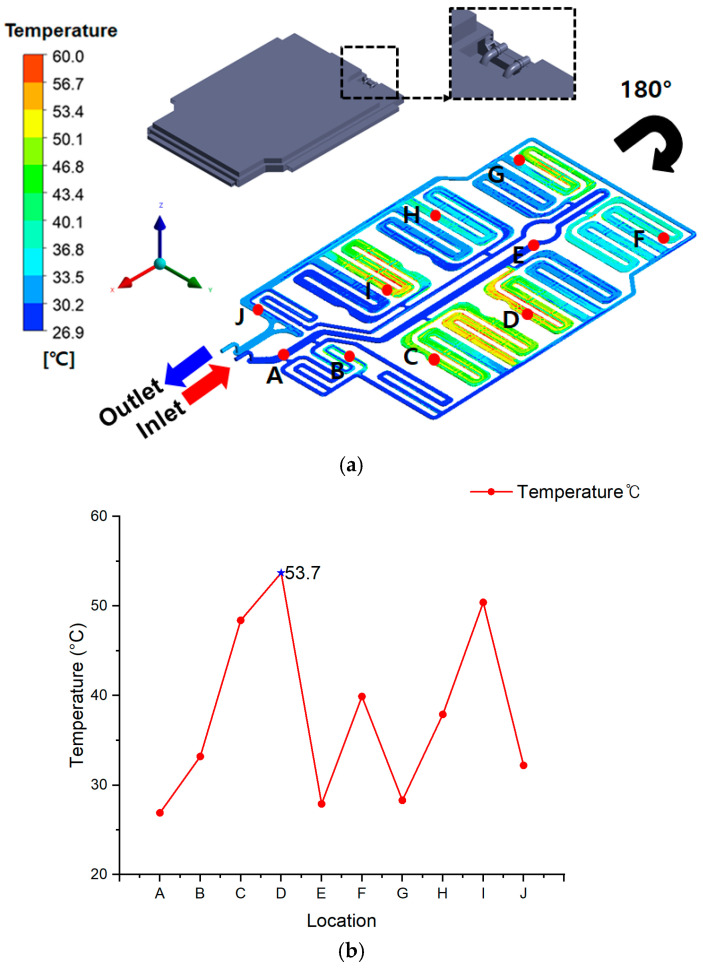
Temperature distribution and coolant performance of the battery pack case with flame-retardant design under full-cell thermal runaway simulation: (**a**) schematic of the cooling channel and monitoring points (A–J), presented with updated orientation and inlet/outlet direction for clarity; (**b**) temperature profile at points A–J, showing the maximum temperature of 53.7 °C at point D (U-shaped bend section with low local velocity and high module density) and the second-highest at point I (opposite U-shaped bend section with accumulated heating), confirming that BMS-controlled coolant circulation and flame-retardant synergy effectively suppressed heat propagation.

Figure 10a illustrates the coolant-flow schematic with temperature monitoring points (A–J), including the inlet and outlet positions. The model was rotated by 180° to match the actual coolant-flow orientation and the coordinate system applied in the CFD analysis. The corresponding temperatures measured at each point are summarized in Table 5, ranging from 26.9 °C to 53.7 °C, with an average of approximately 40.2 °C.

Despite imposing an extreme thermal-runaway heat source of 1107 °C across all cell regions, the combined action of the embedded cooling channel and the silicone-based flame-retardant layer effectively suppressed heat propagation throughout the BPC.

As shown in Figure 10b, the highest temperature (53.7 °C) occurred at point D, which corresponds to a U-shaped bend in the coolant path. This region is characterized by reduced local velocity and prolonged residence time, resulting in a lower convective heat-transfer coefficient. Additionally, point D is located adjacent to a densely packed battery-module region, which increases internal heat accumulation. These combined effects generated the peak temperature observed in the system.

In contrast, the coolant entering through the inlet first travels along a straight segment, where the flow remains uniform and relatively fast. Consequently, upstream monitoring points A–C maintain low and stable temperatures. After passing through the curved sections, the coolant accelerates again near the outlet, enhancing convective heat transfer. Therefore, the outlet-side temperatures remain lower than those at point D, even though the coolant has absorbed heat during circulation.

The second-highest temperature was observed at point I, located at another U-shaped bend in the lower section of the cooling path. Although point I experienced cumulative heating from upstream regions, improved flow redistribution and reduced residence time resulted in a lower peak temperature compared with point D.

Overall, points D and I exhibited elevated temperatures due to:

1. Flow stagnation and weakened convection inherent to U-shaped bends;
2. Localized heat concentration around densely packed module zones.

Nevertheless, the stagnation effect was most severe at point D, making it the hottest location in the system. All other points (A, B, C, E–J) remained near 40 °C, indicating stable thermal behavior under extreme heat-source conditions.

These results confirm that the synergy between coolant circulation (BMS-controlled) and the silicone-based flame-retardant layer provides highly reliable thermal suppression performance, even during full-cell thermal-runaway events. Thus, the flame-retardant EV BPC design ensures robust thermal safety under severe TR scenarios.

### 3.2. Specimen Test Results

Direct flame tests at the specimen level were conducted to evaluate the flame-retardant performance of the EV BPC. In these tests, the front surface of each specimen was exposed directly to a butane-torch flame at approximately 1100 °C (±10%) for 300 s. Thermocouples were installed on the unexposed backside of the specimen to monitor the rear-surface temperature response, while the internal furnace environment was simultaneously measured to verify the applied flame temperature.

**Figure 11:**
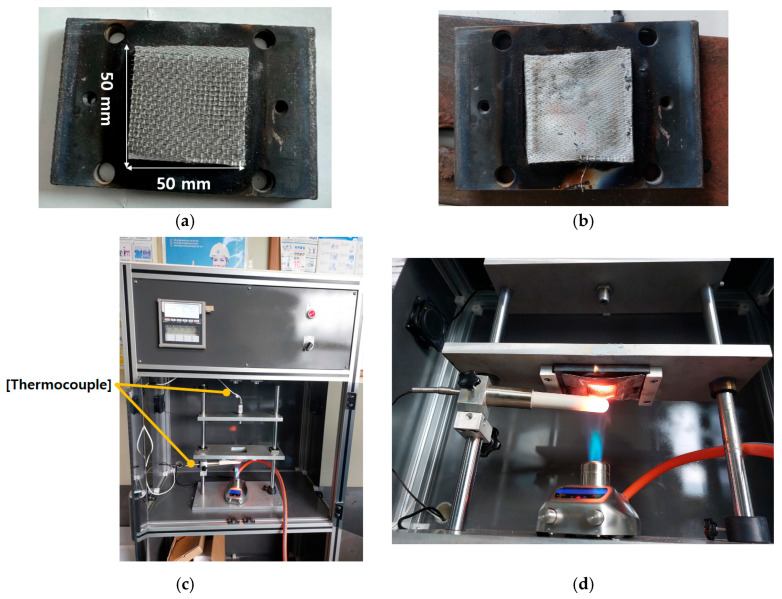
Photographs of the specimen-level fire test: (**a**) specimen before testing; (**b**) specimen after exposure; (**c**) overall test setup with butane torch flame applied; (**d**) close-up view of the direct flame exposure during the test.

Figure 11 presents the visual outcomes of the test. Figure 11a shows the specimen before exposure, and Figure 11b illustrates its condition after 300 s of flame application, clearly indicating charring and localized surface degradation. Figure 11c displays the overall test setup with thermocouples attached to the backside of the specimen, and Figure 11d shows the butane-torch flame being directly applied to the specimen surface during testing.

These observations confirm that the silicone-based flame-retardant material maintained structural integrity under severe direct-flame exposure while limiting heat transfer to the unexposed surface.

Table 6 summarizes the temperature changes over time on the specimen’s unexposed surface and inside the furnace. At the initial stage (0 s), the temperature of the unexposed surface was approximately 160 °C. This elevated initial value is interpreted as the thermocouple being influenced by surrounding radiant heat before the test began. Immediately after flame application, the strong airflow produced by the burner caused a momentary cooling effect around the thermocouple sensors, temporarily lowering the measured temperature to about 65 °C. However, this drop does not represent an actual decrease in the specimen’s temperature; rather, it reflects the response characteristics of the thermocouple under forced convection.

After this transient effect, the temperature steadily increased, reaching approximately 160 °C at 300 s, confirming that the silicone-based flame-retardant material effectively limited heat penetration from the front surface. Meanwhile, the furnace temperature remained stable at approximately 1100 °C (±10%), verifying that consistent test conditions were maintained throughout the experiment.

**Figure 12:**
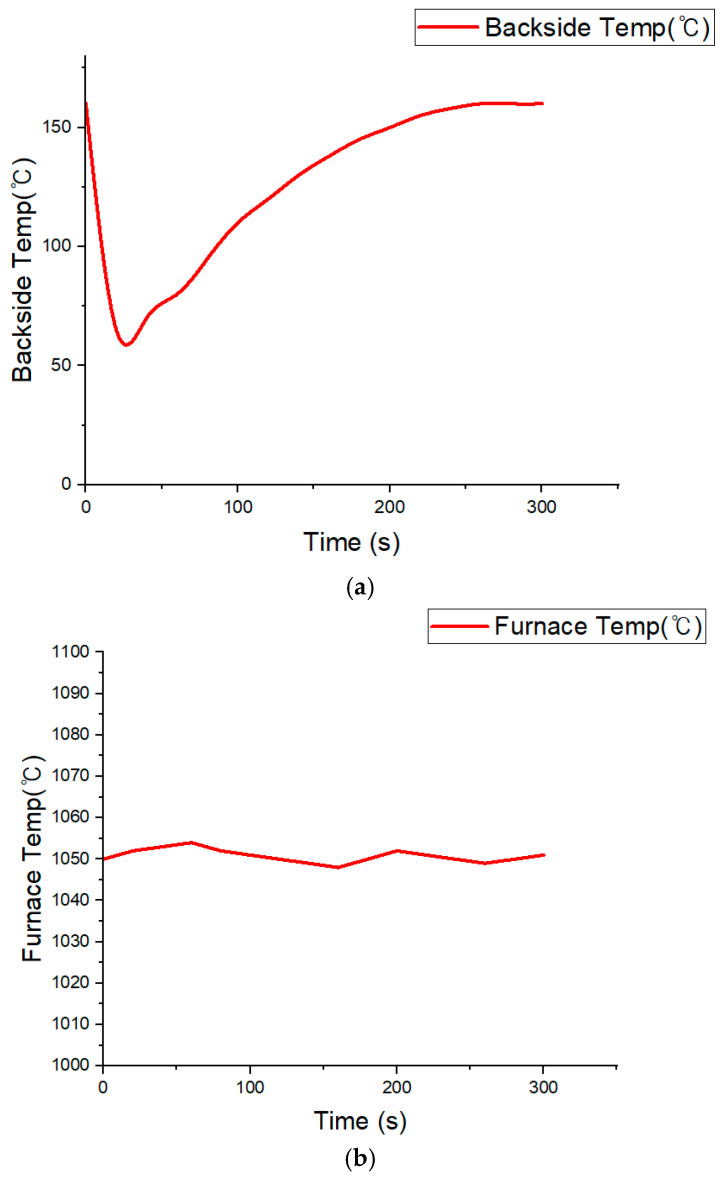
Temperature histories obtained from the specimen-level fire test: (**a**) unexposed surface temperature evolution of the specimen, showing an initial drop to ~65 °C followed by recovery to ~160 °C after 300 s; (**b**) furnace flame temperature maintained within ~1050 °C throughout the 300 s exposure.

Figure 12 shows the temperature history curves based on the data in Table 6. As shown in Figure 12a, the temperature on the unexposed surface initially decreased sharply due to burner-induced airflow, then gradually increased and stabilized near 160 °C. This behavior demonstrates that the silicone-based flame retardant effectively impeded direct heat transfer from the flame. Figure 12b confirms that the furnace temperature remained constant at approximately 1100 °C (±10%) during the entire exposure period.

This behavior can be approximated using a lumped heat-capacity model. The heat flux from the external flame can be expressed as follows:

(1) $$q^{\prime \prime }=h\left(T_{g}-T_{s}\right)+\varepsilon \sigma (T_{g}^{4}-T_{s}^{4})$$

where T_g_ is the gas temperature, T_s_ is the specimen surface temperature, h is the convective heat transfer coefficient, ε is the emissivity, and σ is the Stefan–Boltzmann constant.

The temperature change under transient conditions can be expressed as:

(2) $$\frac{dT}{dt}=\frac{h_{eff}A}{\rho_{cP}V}\;(T_{g}-T)$$

The transient temperature is then given by

(3) $$T\left(t\right)=T_{g}+\left(T_{i}-T_{g}\right)e^{-t/\tau }$$

where the time constant (Ƭ) is defined as:

(4) $$\tau =\left(\frac{{\rho c}_{P}V}{h_{eff}A}\right)$$

Equations (1)–(4) show that the temperature-rise behavior of the unexposed surface follows a first-order lag response, which agrees with the cooling–reheating pattern observed in Figure 12a. This confirms that the proposed flame-retardant design not only suppresses instantaneous ignition but also ensures stable thermal regulation and preserves mechanical integrity under prolonged flame exposure.

Importantly, the backside temperature remained below 160 °C, which is lower than the commonly reported onset temperature for thermal-runaway initiation in lithium-ion cells (≈160 °C) as noted by Lee et al. [55]. Although localized charring occurred on the silicone-based flame-retardant layer, no structural damage or cracking was observed on the rear metal substrate, demonstrating reliable high-temperature durability.

### 3.3. Upper-Case Test Results

This section presents the results of the large-scale flame test conducted on the upper case of the EV battery pack. The test was performed in a vertical furnace using methane as the fuel. The upper case was mounted onto an opening measuring 1000 mm × 1000 mm and sealed with ALC blocks. Six thermocouples (TC1–TC6) were attached to the unexposed surface to monitor the temperature. The test conditions were based on the external flame exposure procedures specified in GB/T 31467.3-2015 and ISO 12405-3. The objective of the test was to examine whether the maximum temperature of the unexposed surface could remain below approximately 160 °C, considering recent findings that thermal runaway onset may occur near this temperature [55]. This design target is more conservative than the rear-surface temperature limit of 200 °C commonly adopted in international safety standards and is consistent with the temperature criterion applied in the specimen-level test in Section 3.2. During the test, the furnace temperature exceeded 500 °C approximately five minutes after ignition and remained stable within the range of 520–530 °C for the next 10 min.

This condition differs significantly from the specimen-level test, which applied a direct butane-torch flame of approximately 1100 °C (±10%) under an open-air condition. In the open-air configuration, part of the incident heat was naturally dissipated by strong convection, causing an initial cooling effect around the thermocouples. In contrast, the upper-case test was performed inside a sealed methane furnace, where heat continuously accumulated without any convective losses. Therefore, from the perspective of the external heat flux defined in Equation (1), the 520 °C furnace environment represents a conservative and sustained thermal load, despite its lower temperature, because radiative and convective heat dissipation is fundamentally restricted compared with realistic EV operating conditions in which coolant circulation and natural convection are present.

To clarify this difference in thermal loading, the visual heat input inside the furnace is expressed using the concept of cumulative heat absorption as follows:

(5) $$Q\left(t\right)=\int_{0}^{t}[h\left(T_{g}-T_{s}\right)+\varepsilon \sigma (T_{g}^{4}-T_{s}^{4})]A\;dt$$

where Q(t) is the accumulated heat input and AAA is the exposed surface area. According to Equation (5), although the instantaneous furnace temperature during the upper-case test was approximately 520–530 °C, the cumulative heat input under sealed-furnace conditions can be comparable to, or even more severe than, that of the short-term 1100 °C (±10%) open-air torch test. This is because radiative heat is trapped and continuously accumulated inside the closed masonry furnace, whereas partial convective cooling occurs in open-air conditions. Therefore, the upper-case test environment can be regarded as sufficiently conservative for evaluating the flame-retardant performance of the EV BPC.

**Figure 13:**
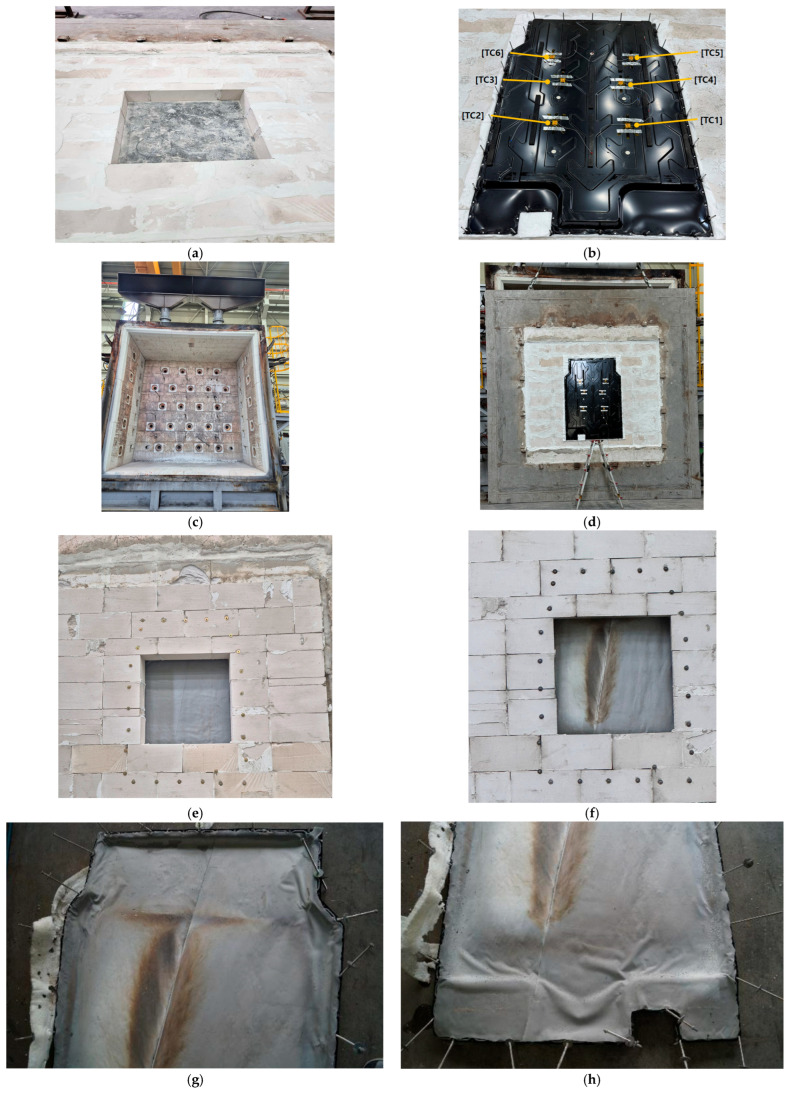
Photographs of the upper-case-level flame test setup and results in a vertical methane furnace: (**a**) Completed masonry with ALC blocks, (**b**) Upper case with six thermocouples attached on the unexposed surface, (**c**) Front view of the large-scale vertical furnace, (**d**) Mounted upper case in the vertical furnace, (**e**) Flame-retardant upper case before the test, (**f**) Flame-retardant upper case after the test, (**g**) Upper case (top view) removed from the ALC block after the test, and (**h**) Upper case (bottom view) removed from the ALC block after the test.

Figure 13 shows the test configuration and the pre- and post-test conditions of the upper case. Figure 13a illustrates the ALC-block opening, while Figure 13b shows the upper case with six thermocouples attached to the unexposed surface. Figure 13c presents the front view of the vertical furnace, and Figure 13d shows the upper case mounted inside the furnace. Figure 13e,f display the unexposed surface before and after the test, and Figure 13g,h present the upper and lower surfaces after removal from the ALC block. No ignition or sustained combustion occurred, and only minor localized soot marks were observed. No structural damage was detected, confirming that the silicone-based flame-retardant layer maintained both combustion resistance and mechanical integrity.

**Figure 14:**
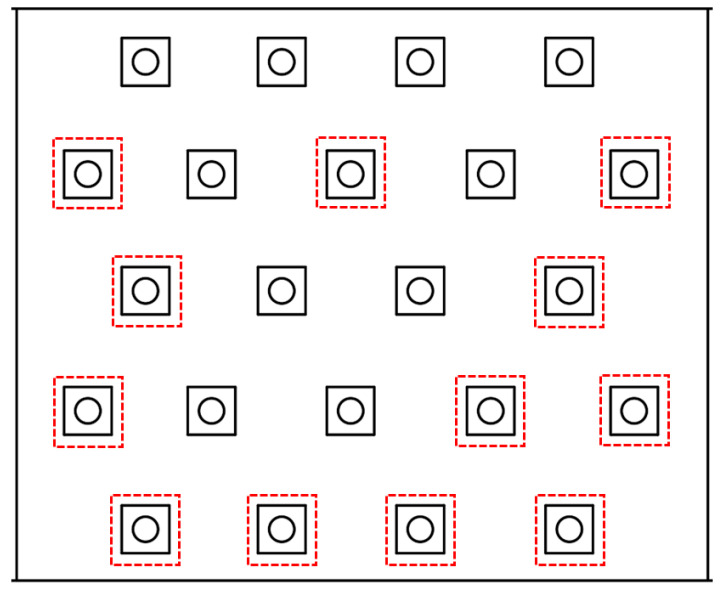
Burner layout of the methane furnace showing the burner outlets used in the test (highlighted in red).

Table 7 summarizes the furnace temperature history, showing that the temperature exceeded 500 °C after approximately five minutes and stabilized in the range of 520–530 °C throughout the 15 min exposure. Table 8 lists the unexposed-surface temperatures measured at six thermocouple positions (TC1–TC6), where the maximum value reached 149.7 °C, thereby satisfying the acceptance threshold of ≤150 °C, which is stricter than the 200 °C limit used in the specimen-level test. This lower threshold is justified because temperatures above ~160 °C are known to accelerate thermal-runaway onset in cells [55], and because coolant-based thermal protection cannot be implemented in large-scale furnace testing environments. The burner layout used in the methane-furnace test is shown in Figure 14, which illustrates the spatial distribution of burner outlets that define the applied flame-heating pattern.

**Figure 15:**
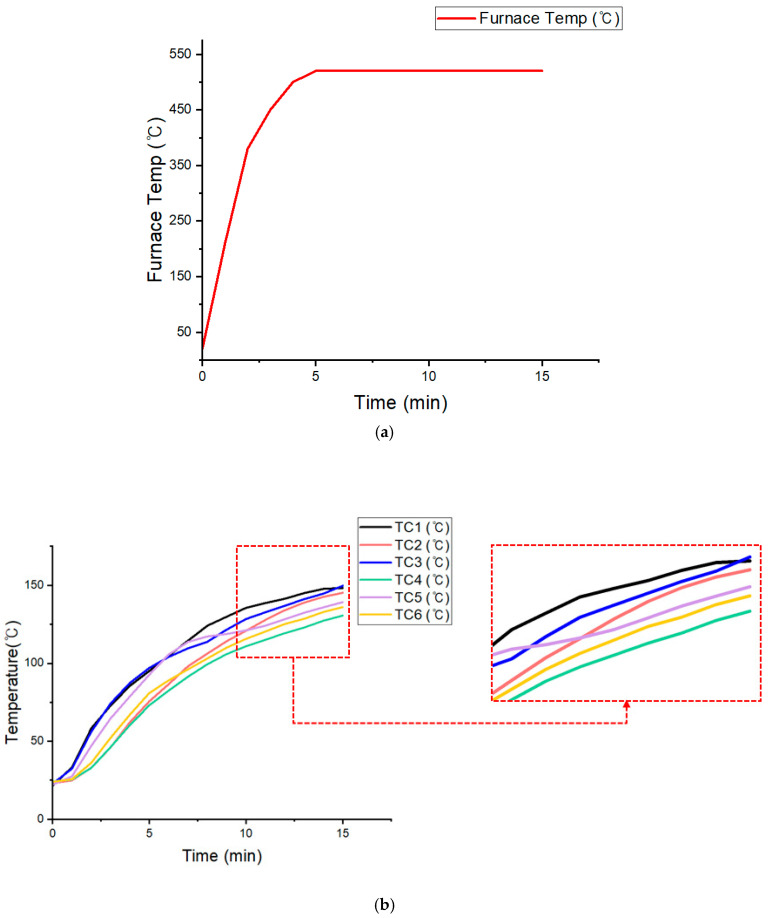
Temperature histories from the upper-case fire test: (**a**) furnace temperature increased to ~530 °C within 5 min and remained stable during the 15 min exposure; (**b**) unexposed surface temperature measured by six thermocouples (TC1–TC6), gradually rising from ~22 °C to a peak of 149 °C, thereby meeting the acceptance criterion (≤150 °C).

Figure 15 provides the corresponding time–temperature curves. Figure 15a shows that the furnace temperature remained stable near 530 °C, while Figure 15b shows that all six unexposed-surface temperature traces increased gradually yet remained below 149.7 °C for the entire duration, meeting the acceptance criteria specified in GB/T 31467.3-2015 and ISO 12405-3.

In practical industrial applications, most EV battery pack enclosures rely on PU-based or ceramic-coated flame-retardant layers, which may suffer from smoke generation, toxic by-products, or structural degradation under prolonged heating. In contrast, the silicone-based flame-retardant layer evaluated in this study maintained integrity without ignition, penetration, or swelling even under 520–530 °C furnace exposure for more than 10 min. This indicates that the proposed design provides a comparable or superior level of rear-surface thermal protection relative to existing industrial systems while offering improved environmental and material stability.

Furthermore, the highest measured unexposed-surface temperature of 149.7 °C has significant safety implications. Temperatures above ~160 °C are widely known to accelerate TR onset at the cell level; thus, maintaining the internal surface below this threshold confirms that the present configuration can effectively suppress TR-propagation pathways and provide additional evacuation time in real EV applications. Based on these findings, further improvements may be realized through multilayer thermal barriers or hybrid insulation systems that combine silicone-based materials with low-density ceramic or aerogel components to extend protection duration under more severe fire scenarios.

In summary, the upper case (i) maintained the unexposed-surface temperature below the required 150 °C threshold under 520–530 °C external-flame conditions, (ii) showed no evidence of ignition or structural degradation after exposure, and (iii) satisfied all international safety requirements for external-flame resistance. Accordingly, the silicone-based flame-retardant upper-case design is validated as an effective means to delay thermal-runaway propagation in real EV battery systems, thereby providing additional evacuation time for occupants. The full-scale upper-case evaluation followed standardized furnace heating protocols to ensure consistent thermal exposure conditions.

## 4. Conclusions

This study comprehensively evaluated the effectiveness of a silicone-based flame-retardant layer applied to an EV battery pack case (BPC) in delaying thermal-runaway (TR) propagation. A multi-stage experimental–numerical framework—including specimen-level direct flame testing, upper-case-level furnace testing, and system-level CFD analysis—was established to validate performance across material, component, and system scales.

In the specimen-level test, the unexposed surface temperature remained below ≈160 °C, well under the 200 °C limit typically referenced for rear-surface protection, even when subjected to a butane-torch flame of 1100 °C (±10%). No structural degradation or cracking was observed, confirming the intrinsic flame-blocking capability of the silicone-based flame-retardant layer. In the subsequent upper-case test, the BPC successfully maintained a maximum unexposed-surface temperature of 149.7 °C, satisfying the acceptance criterion of ≤150 °C specified in GB/T 31467.3-2015 and ISO 12405-3. Despite the furnace temperature exceeding 500 °C and stabilizing within the 520–530 °C range for over 10 min, no ignition, penetration, or mechanical failure occurred. These findings demonstrate that the flame-retardant layer not only prevents ignition but also ensures structural integrity under severe, pack-level fire exposure.

CFD analysis further quantified the thermal behavior of the BPC under realistic operating conditions where coolant circulation and natural convection are present. The simulated temperature distribution and heat-transfer trends showed strong qualitative agreement with the experimental results. Importantly, the heat-flux-based formulation indicated that the cumulative thermal load generated in the sealed furnace environment can be comparable to—or more conservative than—the instantaneous high-temperature load of the open-air torch test. This supports the validity of the upper-case test as a stringent evaluation scenario for practical EV safety design.

From an academic perspective, this study extends TR research beyond traditional cell- or module-level investigations by providing direct experimental validation at the BPC scale, supported by a unified experimental and numerical framework. This multi-layered methodology provides a new approach for evaluating EV fire-resistant structures from the material level through the full case assembly. From an industrial standpoint, the silicone-based flame-retardant layer offers a practical alternative to PU-based materials, addressing environmental concerns while delivering robust flame-blocking performance and minimizing toxic byproducts—thereby offering meaningful implications for next-generation EV safety designs.

Future work will incorporate full battery modules into TR experiments to directly validate performance at the pack scale and refine CFD predictions. Additional research will involve repeated flame-exposure scenarios, variable heat source conditions, and full-pack fire-resistance tests to further generalize the findings and support the development of international standards and industrial applications for silicone-based flame-retardant BPC designs.

## Figures and Tables

**Table 1:** Mesh statistics of the full battery pack case model.

Mesh Parameter	Value
Number of Nodes	16,561,219
Number of Elements	46,263,204

**Table 2:** Material properties of coolant (LCC-10).

Property	Value
Density (kg/m^3^)	1070
Specific Heat (J/kg·K)	3600
Viscosity (kg/m·s)	0.0025
Thermal Conductivity (W/m·K)	0.45

**Table 3:** Material properties of battery pack case (BPC) components.

Part	Material	Density (kg/m^3^)	Specific Heat (J/kg·K)	Thermal Conductivity (W/m·K)	Ref.
Cell	–	2630.7	954	Radial: 1.167 Axial: 12.6	[19]
Pad	EPDM	860	1900	0.25	[45]
Upper Case	SGZCUD 60	7900	470	50	[46]
Module	PA6GF30	1360	1500	0.38	[47]
End Plate	AA6061P	2700	900	167	[48]
Side and MTG	AA6082S-T6	2700	900	175	[48]
Plate	AA3003	2730	893	165	[48]
Support Frame	A6N01S-T6	2700	900	165	[48]
Flame-retardant	Silicone-based	130	1200	0.04	[49]

**Table 4:** Summary of the UL 94 V-0 vertical flame test method [54].

Classification	Test Level	Test Method (Orientation)	Exposure Time	Criteria (Pass)	Classification
UL94 V-0	specimen	Vertical Flame	≤50 s (Total)	Self-extinguish ≤ 10 s	UL94 V-0

**Table 5:** Temperature measurement results at monitoring points (A–J) inside the cooling channel.

Location	Temperature
A	26.9
B	33.2
C	48.4
D	53.7
E	27.9
F	39.9
G	28.3
H	37.9
I	50.4
J	32.2

**Table 6:** Time–temperature history of the specimen-level flame test: unexposed surface temperature of the specimen and furnace temperature recorded during 300 s of direct butane torch exposure.

Time (s)	Unexposed Surface Temp (°C)	Furnace Temp (°C)
0	160	1050
20	65	1052
40	70	1053
60	80	1054
80	95	1052
100	110	1051
120	120	1050
140	130	1049
160	138	1048
180	145	1050
200	150	1052
220	155	1051
240	158	1050
260	160	1049
280	160	1050
300	160	1051

**Table 7:** Temperature measurement results of the furnace interior during the upper-case fire test (0–15 min).

Time (min)	Furnace Temp (°C)
0	20
1	210
2	380
3	450
4	500
5	520
6	520
7	520
8	520
9	520
10	520
11	520
12	520
13	520
14	520
15	520

**Table 8:** Detailed unexposed surface temperature measurements recorded by six thermocouples (TC1–TC6) during the upper-case fire test (0–15 min). The highest temperatures at each channel are highlighted in red for clarity.

Time (min)	TC1 (°C)	TC2 (°C)	TC3 (°C)	TC4 (°C)	TC5 (°C)	TC6 (°C)
0	21.6	23.1	22.7	24.2	22.2	23.9
1	33.3	24.9	32.5	25.4	27.5	25.7
2	58.3	33.1	56.2	32.9	47.1	36.2
3	73	46.1	74.3	46.3	64.7	52.3
4	85.6	62.3	87.5	60.5	78.9	67.2
5	95.1	75.6	97.0	73.1	92.7	80.9
6	104.9	86.6	104.1	82.5	105.6	89.1
7	114.5	98.1	109.6	91.4	113.7	96.1
8	124.1	106.2	113.8	99.4	117.2	103.1
9	129.9	114.2	121.6	105.9	118.8	110.1
10	135.6	120.9	128.4	110.9	121.1	115.7
11	138.6	127.8	132.7	115.1	124.1	120.4
12	141.4	134	136.9	119.3	128.2	125.2
13	145.0	138.9	141.1	122.9	132.4	128.5
14	147.7	142.6	144.7	127.3	135.9	132.9
15	148.3	145.2	149.7	130.6	139.2	136

## Data Availability

The original contributions presented in this study are included in the article. Further inquiries can be directed to the corresponding authors.
